# Estimation of intravoxel incoherent motion parameters using low b-values

**DOI:** 10.1371/journal.pone.0211911

**Published:** 2019-02-06

**Authors:** Chen Ye, Daoyun Xu, Yongbin Qin, Lihui Wang, Rongpin Wang, Wuchao Li, Zixiang Kuai, Yuemin Zhu

**Affiliations:** 1 Key Laboratory of Intelligent Medical Image Analysis and Precise Diagnosis of Guizhou Province, School of Computer Science and Technology, Guizhou University, Guiyang, China; 2 Department of Radiology, Guizhou Provincial People’s Hospital, Guiyang, China; 3 Harbin Medical University Cancer Hospital, Harbin, China; 4 Univ Lyon, INSA Lyon, CNRS, INSERM, CREATIS UMR 5220, U1206, Lyon, France; University of Queensland, AUSTRALIA

## Abstract

Intravoxel incoherent motion (IVIM) imaging is a magnetic resonance imaging (MRI) technique widely used in clinical applications for various organs. However, IVIM imaging at low b-values is a persistent problem. This paper aims to investigate in a systematic and detailed manner how the number of low b-values influences the estimation of IVIM parameters. To this end, diffusion-weighted (DW) data with different low b-values were simulated to get insight into the distributions of subsequent IVIM parameters. Then, in vivo DW data with different numbers of low b-values and different number of excitations (NEX) were acquired. Finally, least-squares (LSQ) and Bayesian shrinkage prior (BSP) fitting methods were implemented to estimate IVIM parameters. The influence of the number of low b-values on IVIM parameters was analyzed in terms of relative error (RE) and structural similarity (SSIM). The results showed that the influence of the number of low b-values on IVIM parameters is variable. LSQ is more dependent on the number of low b-values than BSP, but the latter is more sensitive to noise.

## Introduction

Intravoxel incoherent motion (IVIM) imaging is a magnetic resonance imaging (MRI) technique widely used in clinical applications for various organs. It consists in extracting perfusion information from diffusion-weighted (DW) signals [1–3]. In this technique, the attenuation of DW signals at each image voxel is assumed to be caused by both the diffusion of water molecules in tissues and the microcirculation of blood inside blood vessels, which is also called intravascular perfusion or pseudo-diffusion. Pseudo-diffusion information was shown to enable normal and pathological tissues to be distinguished [4–7].

In IVIM imaging, DW signals are first acquired at several b-values, and then a fitting technique is employed to solve a system of equations. Usually, pseudo-diffusion parameter has much higher value than diffusion parameter. This implies that, for a given b-value, the pseudo-diffusion (exponential) term decays much faster than the diffusion (exponential) term. When the b-value is higher (saying higher than 200 s/mm^2^), diffusion plays a dominant role and the contribution of perfusion to DW signals almost disappears [8–14]. Therefore, to accurately estimate perfusion-related parameters, DW signals must be acquired with not only high and but also low b-values (saying smaller than 50 s/mm^2^). Until now, there is not yet international standard on the definition of low or high b-value. Often, at a b-value of about 200, pseudo-diffusion (or perfusion) can be negligible for the estimation of diffusion component. Therefore, all b-values superior to 200 could be considered high. Likewise, at a b-value of about 50, pseudo-diffusion can no longer be negligible. In this case, all b-values inferior to 50 could be considered low. Thus, the notion of low or high b-values is dependent on tissues and applications in question.

IVIM imaging at low b-values is very important [15,16] because IVIM models contain terms that decrease exponentially with the increase of b-values. At high b-values, the variation of IVIM models are relatively slow, meaning that relatively few b-values would be necessary to describe the variation trend. In contrast, at low b-values, the IVIM models have a rapid decreasing, implying that more low b-values would be needed to better describe the variation trend. In addition, low b-value IVIM imaging is essential for organs whose pseudo-diffusion has relatively high values, such as liver [10,17–19], pancreas [20], skeletal muscle [21–23], and kidney [24]. These reported works used low b-values but did not study how the choice of the number of low b-values will influence the results, in particular in the presence of noise.

Once the DW data were acquired, the used fitting method would also influence the parameter estimation accuracy of the IVIM model. Currently, the standard IVIM fitting technique is the nonlinear least squares (LSQ) method [13], which consists of minimizing the difference between the real and predicted DW signals. More recent methods include Bayesian techniques, which were initially proposed by Neil et al. [25]. Unlike the LSQ method, the principle of Bayesian methods is to maximize the posterior probability of IVIM parameters given the observed signal [13,26]. In this kind of methods, the likelihood probability is dependent on the predicted DW signal [14,27,28] that is closely related to the number of low b-values. Freiman et al. tried to improve the IVIM parameter estimation accuracy by using a Bayesian model with spatial homogeneity prior (FBM) to obtain the localized smooth IVIM parameter maps [26]. Orton et al. used a Bayesian fitting method with Gaussian shrinkage prior (BSP) to make outlying estimations be shrunk towards the mean of the region of interest (ROI) [14]. Based on these works, While et al. compared systemically the performance of LSQ and Bayesian methods and showed that Bayesian methods outperformed LSQ methods for IVIM parameter estimation [13]. However, these studies did not consider the influence of the number of low b-values while too few low b-values might ignore pseudo-diffusion components.

In addition, it is well known that DW signals are corrupted by noise [29]. Therefore, the signal-to-noise ratio (SNR) is a factor to be taken into account in the estimation of IVIM parameters.

To account for the above-mentioned problems of low b-value, fitting method and noise, we propose to study in a systematic and detailed manner the influence of the number of low b-values on IVIM parameter estimation by also taking into account the factors of SNR and fitting method. To this end, DW data with different numbers of low b-values and SNRs were simulated to get insight into the distributions of subsequent IVIM parameters. Then, in vivo DW data with different numbers of low b-values and different number of excitations (NEX, also called average times in the following) were acquired. Finally, least-squares (LSQ) and Bayesian shrinkage prior (BSP) fitting methods were used to estimate IVIM parameters under different combinations of low b-values and average times.

## Materials and methods

### Simulations

To assess the joint effects of low b-value, SNR and fitting method on the estimation of IVIM parameters, we simulated the IVIM signals with a bi-exponential model with the influence of noise [14],

$$S_{n}=S_{0}(Fe^{-b_{n}D^{*}}+(1-F)e^{-b_{n}D})+\varepsilon_{n}$$ (1)

where *S*_*n*_ is the signal obtained with the *n*^*th*^ b-value *b*_*n*_, *S*_*0*_ is the signal with *b* = 0, *F* is the perfusion fraction, D^*^ and *D* represent the pseudo-diffusion and diffusion coefficients respectively, and *ε*_*n*_ designates the noise.

For the choice of b-values in the simulation, to account for the influence of low b-values, three b-value distributions were considered according to the number of low b-values (0<b<50 s/mm^2^), namely, the b-value distribution with two low b-values (0,10,20,50,70,100,200,400,600,800 s/mm^2^), the b-value distribution with one low b-value (0,20,50,70,100,200,400,600,800 s/mm^2^), and the b-value distribution without low b-value (0, 50,70,100,200,400,600,800 s/mm^2^). For the choice of noise in the simulation, Gaussian noise was used with three different SNRs of 10, 30 and 50 dB. There are various methods to derive SNR; in this work, SNR was calculated as the ratio of mean signal intensity to standard deviation of noise intensity [8,30]. For the choice of the IVIM parameters, the regions of in vivo thigh images were first selected and the corresponding *F*, D^*^ and *D* at each voxel of the regions were then estimated. The signal at a voxel was finally calculated using Eq (1). Note that S_0_ at each voxel is equal to S_0_ of the corresponding voxel in in-vivo data. Repeating the above calculation for each voxel generates a realistic simulated DW image.

### DW image acquisitions

This prospective study was approved by the Ethics Committee of Guizhou People’s Hospital and written informed consents were obtained from all volunteers. The in vivo data of the human thigh was acquired on 3.0 Tesla MRI scanner (GE Discovery MR750) using a DW echo planar imaging (EPI) sequence within about 3 minutes. The main acquisition parameters are as follows: field of view (FOV) = 40×32 cm, matrix size = 96×128, slice thickness = 8 mm, repetition time (TR) = 6000 ms, echo time (TE) = 61 ms, diffusion gradient pulse duration (small delta δ) = 19 ms, time delay between gradient pulses (big delta Δ) = 29 ms, effective diffusion time = 23 ms (approximately equal to Δ-δ/3), and b-values (for fixed diffusion time) = (0, 10, 20, 50, 70, 100, 200, 400, 600, 800 s/mm^2^). To get different SNRs, DW images of the thigh were acquired 6 consecutive times (i.e. NEX = 6, or 6 acquisitions or still 6 average times). Then, we averaged 2, 3, 4, 5, and 6 acquisitions respectively to obtain different SNR improvements. In the real acquisitions, the noise was estimated by subtracting two acquisitions and calculating the standard deviation of the resulting image [31]. By averaging over N consecutive original images, the SNR improved by $\sqrt{N}$ [30,32]. Different average times or simply averages in what follows, lead to different SNRs.

To investigate the influence of low b-values, the DW images were divided into three groups according to the three different b-value distributions mentioned in the simulation section.

### Estimation of IVIM parameters

The estimation of the parameters in Eq (1) is performed voxel by voxel. For the given *S*_*n*_, *S*_*0*_ and *b*_*n*_, the IVIM parameters *D*, D^*^ and *F* are estimated respectively using the LSQ or BSP.

#### LSQ-based method

The family of LSQ methods consists of adjusting the parameter values of the bi-exponential model (Eq (1)) by minimizing the sum of squared error between the fitted signal and acquired signal, thus yielding the estimates of IVIM parameters. The objective function of the minimization problem is given by:

$$\min\sum_{n=0}^{N}{\left(S_{n}-S_{n}^{'}\right)}^{2}$$ (2)

where *S*_*n*_ represents the acquired signal, $S_{n}^{'}$ the fitted signal provided by the model after LSQ fitting, and *N* the number of b-values.

The most commonly used LSQ methods are full least squares fitting and segmented least squares fitting [6,9,33]. With the full least squares fitting method, all the parameters of the IVIM model are fitted at the same time in a single whole process. This method is simple and easy to implement, but the estimates of the parameters are not accurate because of the interaction between diffusion and perfusion components on signals. To improve the estimation accuracy of fitting parameters, the segmented least squares fitting method adopts a step-by-step fitting mechanism. In the first step, assuming that the signal of perfusion component at high b-values (b>200 s/mm^2^) is completely attenuated to zero, the parameter *D* is fitted using the mono-exponential model

$$S_{n}=S_{0}e^{-b_{n}D}$$ (3)

Then, the parameter *F* is estimated using the extrapolated signal of the fitted mono-exponential model as follows [6]:

$$F=(S_{0}-\mathrm{intercept})/S_{0}$$ (4)

where “intercept” is the fitted *S*_*0*_ in Eq (3). In the second step, after fixing *D* and *F* estimated in the first step, the remaining parameters of the bi-exponential model expressed in Eq (1) are finally estimated. Since *D* and *F* are estimated under the assumption that the signal of perfusion component at high b-values is almost attenuated to zero, such estimation will be biased if the assumption is not strictly satisfied. In other words, to improve the fitting accuracy of IVIM parameters, images acquired with low b-values may be necessary.

**Fig 1:**
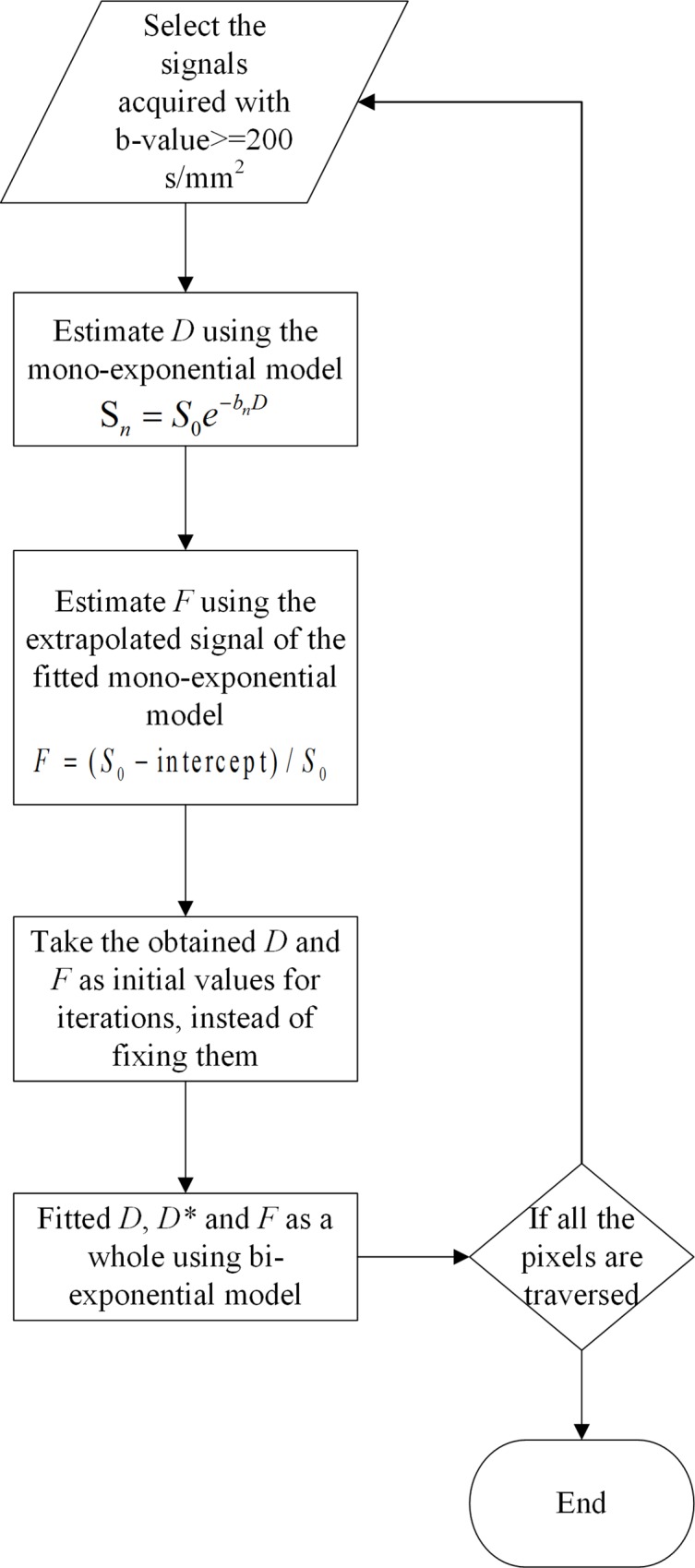
The flow chart of LSQ fitting.

In the present work, we combined the full and segmented least squares methods to fit the IVIM parameters, as illustrated in the fitting process scheme of Fig 1. We first used the segmented least squares method (Eqs (3) and (4)) to obtain *D* and *F* that serve as initializations. The final parameter values of *D F* and D* were then estimated simultaneously using the full least squares method. To stabilize the fitting when computing voxel-wise estimates, the following constraints were chosen as done in [14]: 0.0005 < *F* < 0.9995; 0.045 < *D* < 18 (x 10^−3^ mm^2^/s); 0.34 < D* < 1000 (x 10^−3^ mm^2^/s).

#### BSP-based method

Another fitting method we used is the BSP method proposed by Orton et al. It improves the quality of parameter estimates using a Gaussian shrinkage prior. The basic idea of BSP is to maximize a joint posterior probability of IVIM parameters, given the observed data:

$$p(\theta_{1:M},\mu ,\Sigma_{\mu }|y_{1:M})\propto \prod_{i=1}^{M}p(y_{i}|\theta_{i})p(\theta_{i}|\mu ,\Sigma_{\mu })$$ (5)

where *θ* is a vector of three IVIM model parameters (*f*, *d* and d*) in transformed form with *f* = log(*F*)−log(1−*F*), *d* = log*D* and d* = logD*, *M* is the number of voxels, *μ* is a vector containing the mean value of the transformed parameter over all voxels, ∑_*μ*_ is a 3 * 3 covariance matrix of *μ*, and *y*_*i*_ represents the signal at the current voxel. The likelihood function *p*(*y*_*i*_|*θ*_*i*_) is a multivariate conditional probability that takes the form [14]:

$$p(y|F,D,D^{*})\propto {\left[y^{T}y-{\left(y^{T}g\right)}^{2}/(g^{T}g)\right]}^{-N/2}$$ (6)

where *g* is the expected signal vector obtained with different b-values without error term normalized by the signal *S*_0_ at *b*_0_, and *N* is the number of b-values. The prior function *p*(*θ*_*i*_|*μ*,∑_*μ*_) subjects to a multivariate Gaussian distribution, the formulation of which is [14]:

$$p(\theta_{i}|\mu ,\Sigma_{\mu })=|2\pi \Sigma_{\mu }{|}^{-1/2}\exp(-\frac{1}{2}{\left(\theta_{i}-\mu \right)}^{T}\Sigma_{\mu }{}^{-1}(\theta_{i}-\mu ))$$ (7)

**Fig 2:**
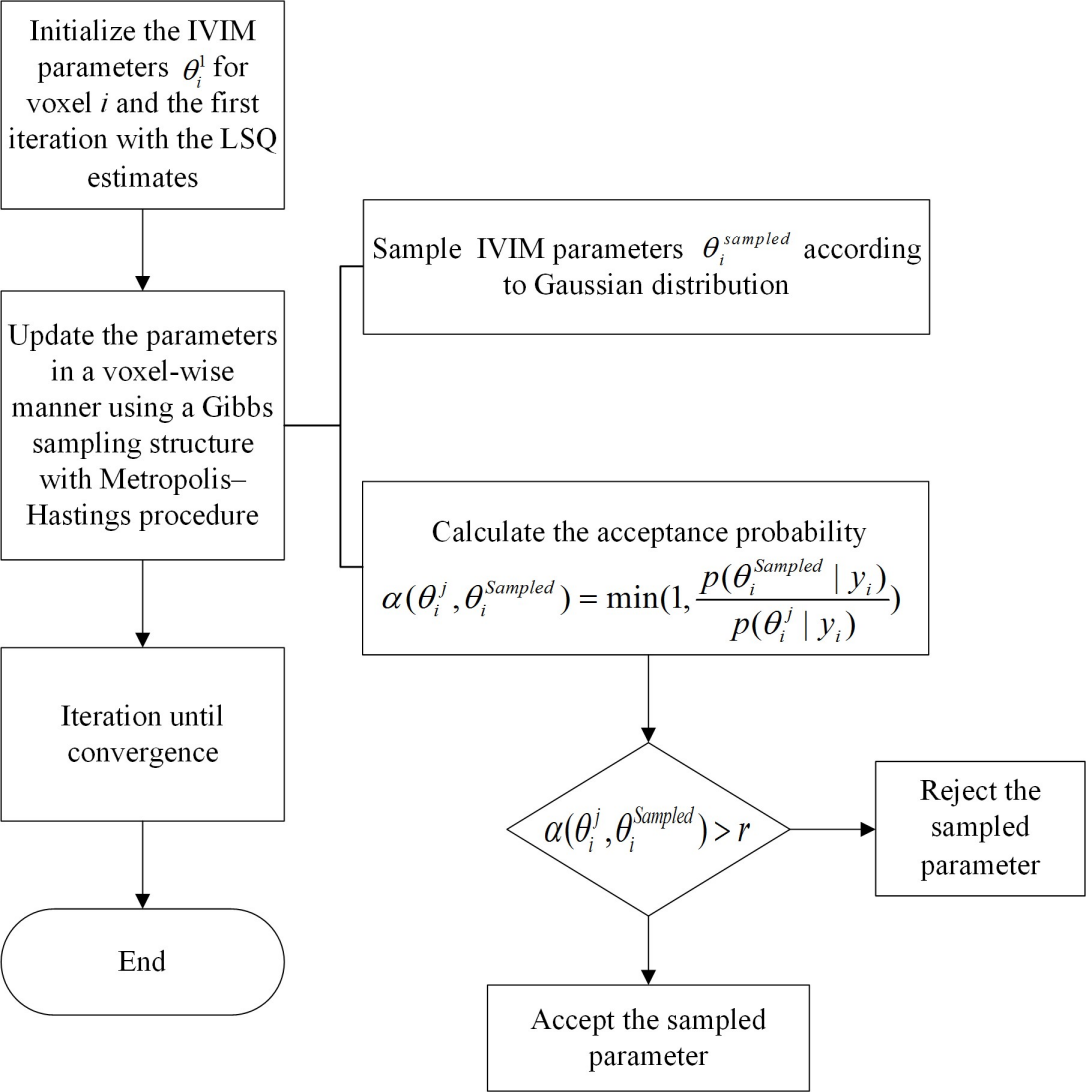
The flow chart of BSP inference. *r*~*U*, *U* is a uniform distribution between 0 and 1.

The iteration process for fitting was realized by Markov chain Monte Carlo (MCMC) algorithm that is illustrated in Fig 2 [14].

### Quantitative analysis

The IVIM parameters obtained using different combinations of b-values, SNRs, and fitting methods were compared using several criteria. For the simulation results, due to the existence of the ground-truth, the relative error (RE) and the structural similarity (SSIM) for IVIM parameters were calculated at each voxel. RE is defined as

$${\mathrm{RE}}_{i:n}=\frac{\left|\left(T_{i}-P_{i}\right)\right|}{T_{i}}\times 100$$ (8)

with *T*_*i*_ and *P*_*i*_ representing respectively the ground-truth and predicted IVIM parameters at the *i*^*th*^ voxel.

SSIM was calculated to measure the similarity between estimated and ground-truth parameter maps with the value range between 0 and 1. The closer the SSIM value is to 1, the closer the estimated parameter map is to the ground-truth.

Finally, the differences in RE and SSIM between different combinations were compared using two-sample t-test and all analyses were performed using Matlab (R2013b).

## Results

### Estimation of IVIM parameters for simulated images

**Fig 3:**
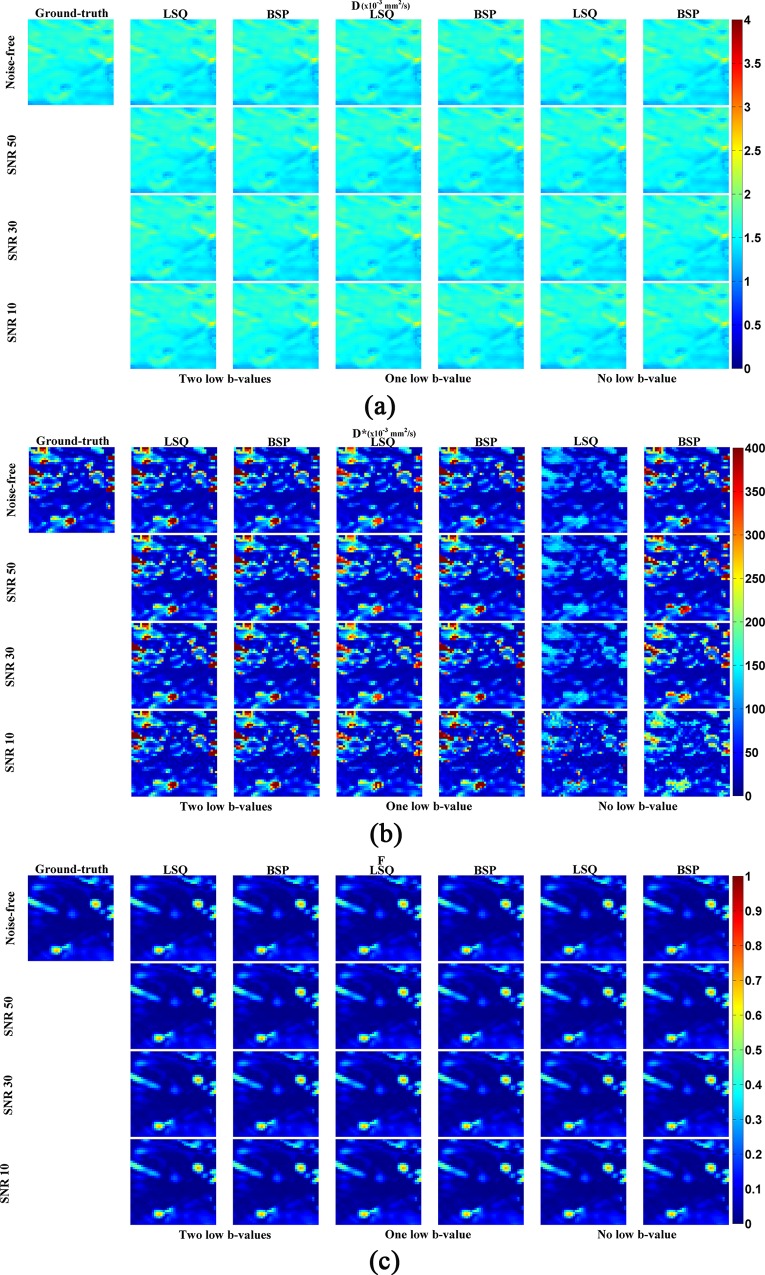
Estimation of IVIM parameters on simulated data with different b-value distributions, SNRs and fitting methods. “Two low-b-values”: the b-values of 10 and 20 s/mm^2^ are included in the fitting. “One low b-value”: the b value of 20 s/mm^2^ is included in the fitting. “No b-value”: all the b-values used in the fitting are greater or equal to 50 s/mm^2^. In the following figures, the same expression is used.

To compare quantitatively the joint effects of low b-value, SNR and fitting method on the estimation of IVIM parameters, several simulations were performed with different combinations. The IVIM parameters including *D*, D* and *F* obtained on the simulated data are shown in Fig 3. We observe that, in almost all the situations, *D* and *F* maps estimated using both LSQ and BSP are very close to the ground-truth. In contrast, the estimation of D* is greatly dependent on imaging conditions, especially on the number of low b-values, noise levels and fitting methods. When the number of low b-values is enough (two low b-values are enough in our present study), the estimated D* is not sensitive to noise level or fitting method, which implies that both LSQ and BSP allow estimating accurately D* map and are not influenced by noise (2^nd^ and 3^rd^ columns in Fig 3B). However, when the number of low b-values was decreased, D* was underestimated by LSQ at some voxels, especially for the case without low b-values (4^th^ and 6^th^ columns in Fig 3B). As to the D* map obtained by BSP, its variation with the number of low b-values was not obvious, except in the case without low b-values or with low SNR. In the latter case, the estimation accuracy of BSP in D* was decreased but still much better than LSQ (5^th^ and 7^th^ columns in Fig 3B.

**Fig 4:**
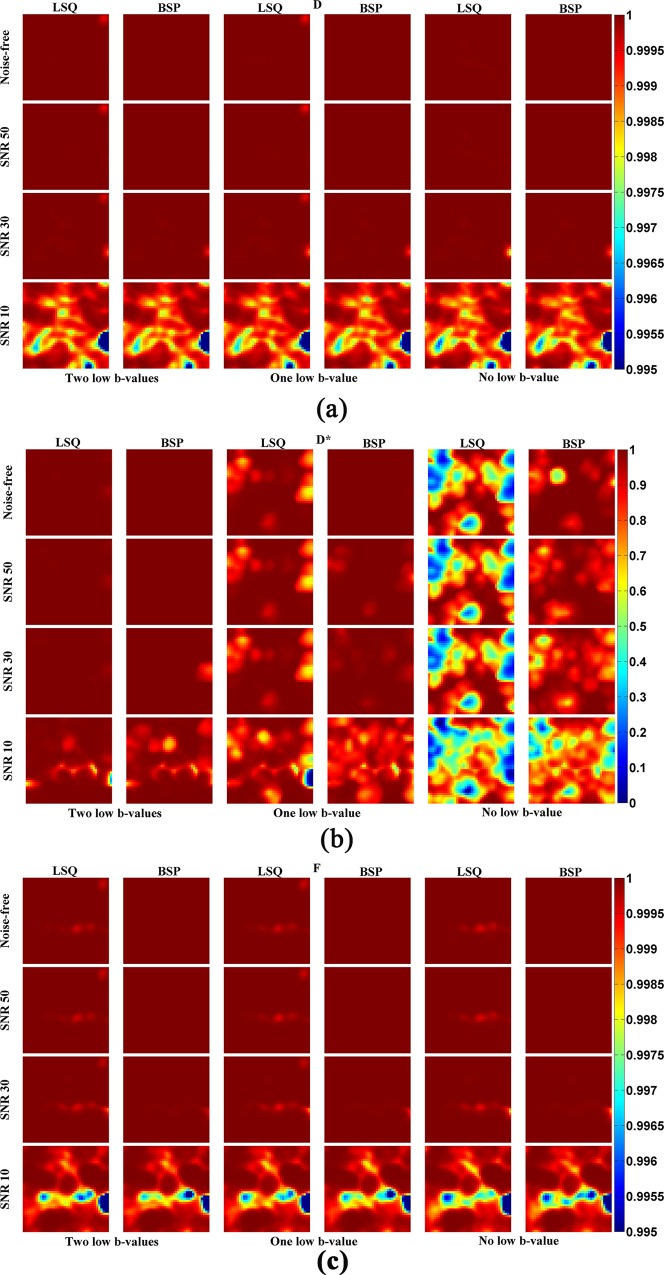
SSIM maps of the estimated IVIM parameters for different combinations of low b-values, SNRs and fitting methods.

**Fig 5:**
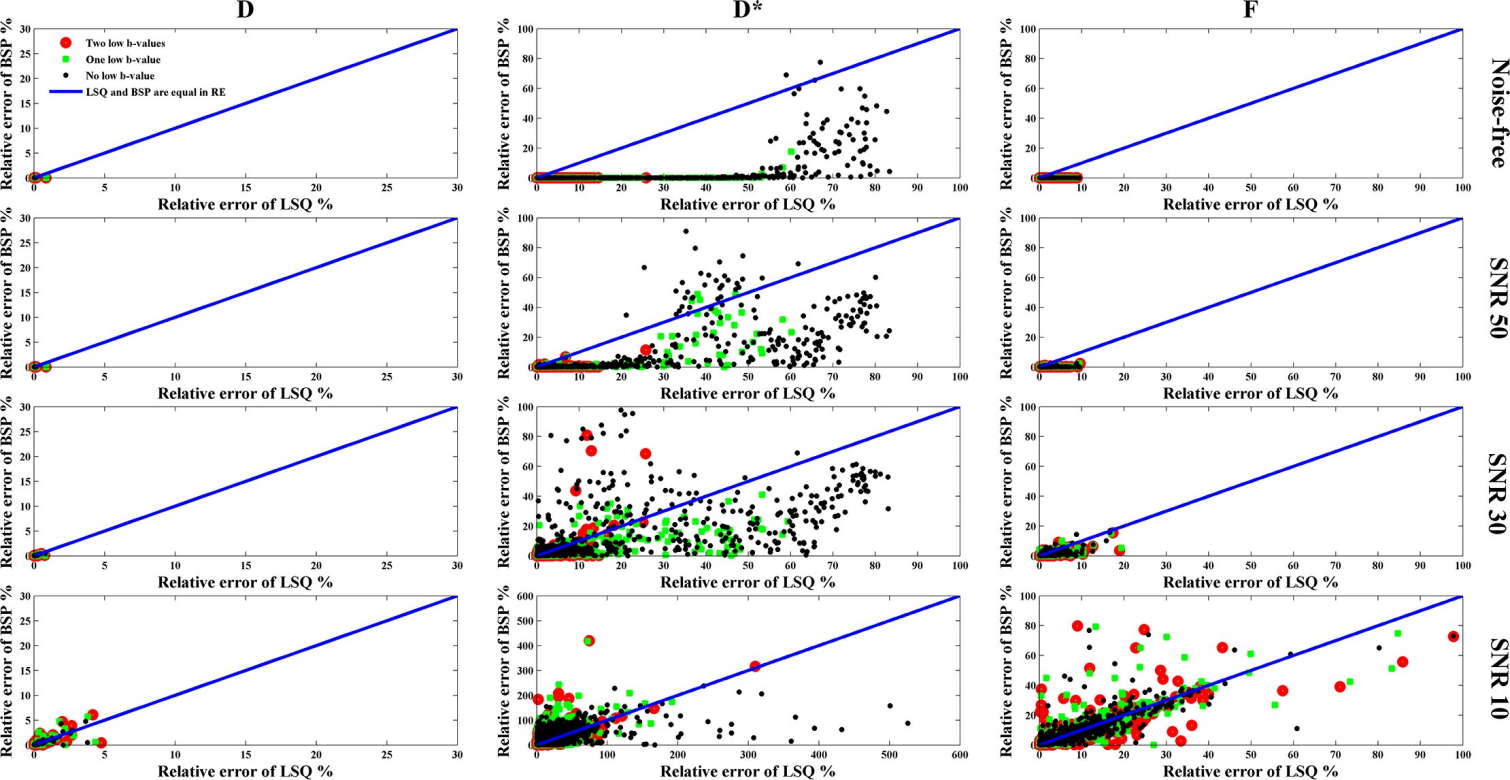
Relative error (RE) of the estimated IVIM parameters for different combinations of low b-values, SNRs and fitting methods. The coordinates of each point represent the RE of each voxel obtained using different fitting methods.

To quantitatively evaluate how the estimated IVIM parameter map is close to the ground-truth, in Fig 4 and Fig 5 are shown the SSIM and RE maps for different combinations of low b-values, SNRs and fitting methods. The SSIM maps of *D* or *F* generated by LSQ and BSP were very close (P>0.1) although the SSIM obtained by BSP was a little higher than that by LSQ. As to D*, its SSIM map obtained by BSP was better than that derived from LSQ (P<0.001). Note that for the SSIM map of D* obtained by LSQ, the less the low b-values, the worse the SSIM map. At the same time, the SSIM map obtained by BSP was more sensitive to the noise level whereas the influence of low b-values was not significant. The RE map shows the same results; the IVIM parameters estimated by BSP was more accurate than LSQ, especially in the case of few low b-values involved.

### Estimation of IVIM parameters for in vivo images

**Fig 6:**
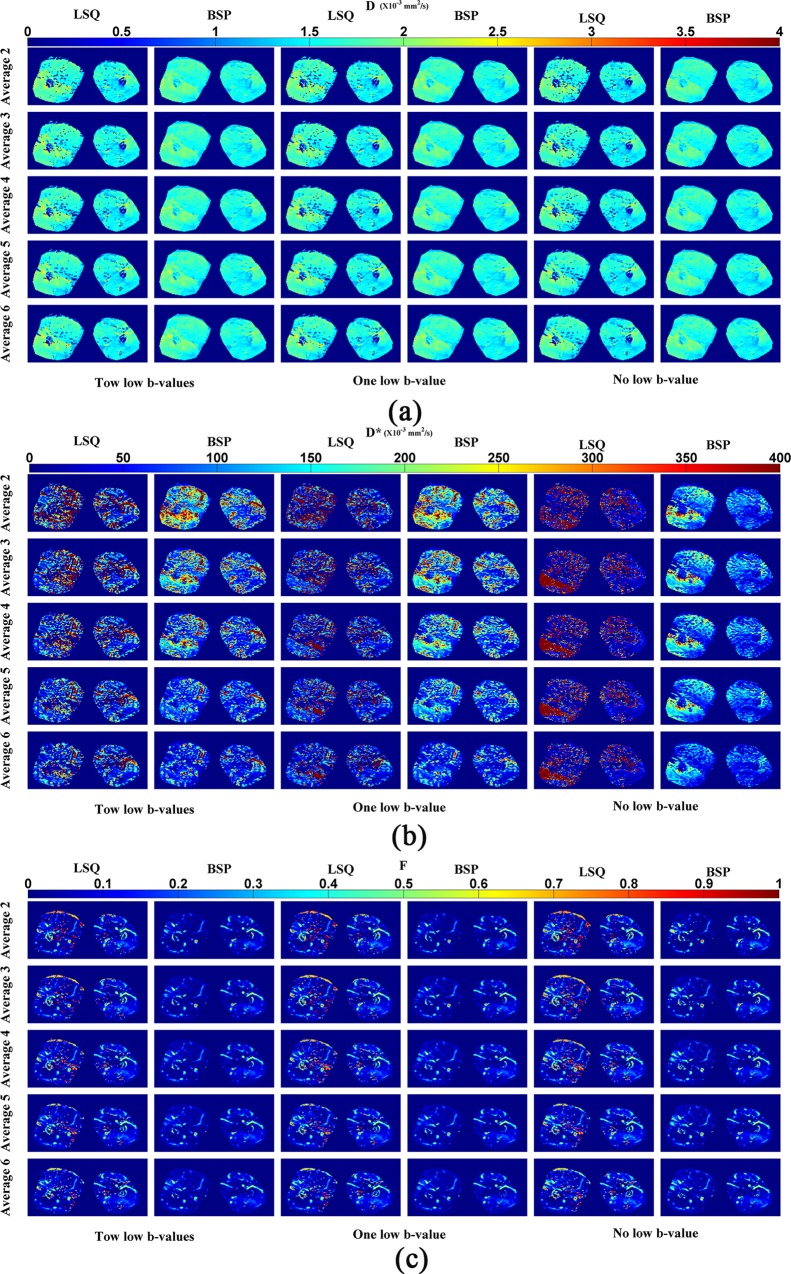
IVIM parameter maps of in vivo thigh for different combinations of low b-values, average times and fitting methods. (a) *D*. (b) D*. (c) *F*. Different averages correspond to different SNRs: 2 averages (SNR = 26 dB), 3 averages (SNR = 37 dB), 4 averages (SNR = 46 dB), 5 averages (SNR = 59 dB), 6 averages (SNR = 65 dB).

The IVIM parameter maps of the in vivo thigh are shown in Fig 6. The SNRs corresponding to 2, 3, 4, 5 and 6 averages represents 26, 37, 46, 59 and 65 dB respectively. All the parameter maps obtained by BSP were clearer than those by LSQ. Concerning the effects of noise and low b-values on the parameters estimation, results similar to the case of simulation were observed. Low b-values and SNR had relatively limited influence on *D* or *F* map with respect to D* map in which the impact of low b-values and SNR was obvious. As the average times were increased, SNR increased and the D* maps obtained by both BSP and LSQ became cleaner due to the reduction of erroneous heterogeneity. With the use of low b-values, the D* map generated by LSQ became cleaner as the average times increases, but such change was much less important when using BSP. If using two low b-values, the latter resulted in poor D* maps in the case of averaging 2, 3 and 4 times. When averaging 5 and 6 times, the D* maps obtained by BSP were much clearer than those obtained by LSQ. When using one low b-value, the results were close to the case of using 2 low b-values, but the erroneous heterogeneity was slightly increased. BSP generated a superior D* map when the average times was set to 6. With the decrease of the number of the low b-values, the D* values of most voxels obtained by LSQ were obviously reduced, which agrees with the results in the simulation study. In this case, some voxels tended to exhibit extreme D* values (part of the red region with high D* values, estimated D*>800 x10^-3^ mm^2^/s), and such results were not obvious when using BSP.

## Discussion

To investigate quantitatively IVIM imaging at low b-values, we have chosen to take also into account the influence of fitting method and SNRs. The investigation of the joint effect of low b-value, SNR and fitting method using both simulation and in vivo thigh images showed that the estimation of *D* and *F* was not influenced greatly by the imaging conditions and fitting methods. However, D* estimated using both LSQ and BSP depends greatly on the number of low b-values used or noise level.

According to the simulation results, the estimation accuracy of D* using LSQ is significantly degraded and the values of D* are underestimated when the number of low b-values is reduced. This finding is consistent with that reported by Cohen et al [10] in which only a fixed SNR was considered. When varying the SNR, the variation of D* is not evident except when SNR is very low. This implies that the performance of LSQ method is much more sensitive to the number of low-b values than to SNR. In contrast, the dependence of BSP performance on the number of low b-values is less strong with respect to LSQ. This also explains why for any number of low b-values, the estimation accuracy of BSP for D* was better than LSQ when the SNR was high enough. However, if SNR is too low, the performance of BSP was not good either, its degradation also depending on the number of low b-values. When using one or two low b-values, we obtained results similar to those of While [13], namely, BSP has better performance than LSQ in the estimation of IVIM parameters. In the case there are no low b-values (zero low b-value), the estimation accuracy of D* was seriously affected by noise. These results are different from those reported in the work of While, in which it was concluded that the Bayesian methods consistently outperform the LSQ-based ones even when the SNR is very low.

The above findings can be explained by the difference of mechanism between LSQ and BSP. LSQ aims to minimize the difference between real and fitted signals through solving a system of equations. If the number of low b-values is not sufficient, the contribution of high-perfusion components to the fitted signals is attenuated to about zero, which results in estimation errors for perfusion coefficient. To deal with this problem, the higher the perfusion parameter value, the more low-b-values required. Thus, the performance of LSQ method relies more on the number of low b-values. In contrast, the nature of BSP is to maximize the posterior probability of parameters given the observed data, which is calculated by multiplying the prior and likelihood probabilities. Since the likelihood and subsequently the predicted signal is related to low b-values, D* estimated by BSP still depends on the low b-values. Nevertheless, such dependence will be reduced by the priori probability, the shrinkage property of which is so strong that it can shrink the outliers toward the mean of ROI. As a result, low b-value dependence is less strong in BSP than in LSQ.

Concerning the influence of SNR, our results suggest that the estimation accuracy of BSP for D* is more sensitive to noise; the fewer the number of low b-values, the more obvious the effect. This phenomenon can also be explained by the nature of Bayesian method. Although the prior distribution can reduce the dependence of BSP on the number of low b-values, it can, however, be corrupted heavily by noise if SNR is low. In such case, the superiority of prior probability is decreased and consequently the performance of BSP on the pseudo-diffusion parameter estimation degrades, especially when SNR is low as well as low b-values are absent.

With regard to the results on in vivo data, they were somewhat different from those reported by Cohen et al [10]. When the number of low b-values were not sufficient, LSQ generated lower D* values at most voxels with respect to the case of using a sufficient number of low b-values, which confirms the findings of Cohen et al [10], except the fact that, at some voxels, the estimated D* tended to have extreme values. This is because, with LSQ, parameter estimation depends greatly on the selection of initial values. When pseudo-diffusion parameter has relatively high values, the influence of perfusion component on the predicted signals is limited, and different D* may generate in this case similar predicted signals and residuals. Therefore, if perfusion faction *F* is low, the contribution of D* values to signal residual is limited, and the results are easier to tend to be extreme in the process of iteration if the selection of initial values is not appropriate. However, such results are not obvious when using BSP.

As to the influence of SNR on in vivo data estimation, when temporal average times are increased, the outlying estimation of D* generated by BSP or LSQ was reduced and the parameter map became clearer. These results were slightly different from those of simulation. The reason is that the IVIM model used in the simulation totally conformed to the ground-truth whereas the IVIM model applied on in vivo data was approximate [7,34–36]. Moreover, the noise added in the simulation data was strictly Gaussian distribution while, in real data cases, noise did not conform to exactly an analytical noise model.

Finally, in the present study, we have used an IVIM acquisition protocol on clinical GE MRI machine, in which b-values are varied by varying gradient strength with fixed δ and Δ. However, the mathematical formula of b-values is a function of three variables: gradient strength, diffusion gradient duration (δ) and interval between pulses (Δ). So, there are mathematically many possibilities of varying b-values. It would then be interesting in the future to vary b-values through varying diffusion time while fixing gradient strength. Since diffusion time is approximately equal to Δ-δ/3, one can vary either Δ with fixed δ or δ with fixed Δ, or vary both of them. Obviously, for fixed gradient strength and δ, changing diffusion time gives rise to different b-values and thus different DW signals, the estimation of IVIM parameters will be impacted. Such estimation will however not be trivial, because it depends on the unknown microstructure of the organ under investigation and on how to choose diffusion time, IVIM model, and fitting technique while accounting for noise influence. For instance, when gradient strength and δ are fixed, too long diffusion time will lead to too big b-values and consequently too important signal attenuation, which may cause too small signal-to-noise ratio (SNR). Microscopically speaking, long diffusion times will leave water molecules to have more chances to encounter obstacles such as cell membranes and fibers, which may lower water diffusion coefficient [37]. In the opposite case, too small diffusion time (with fixed gradient strength and δ) may make it difficult to achieve the desired low b-values, because the latters highly depend on MRI hardware and software and instabilities in gradient amplifiers may render low b-values unreliable [38]. Likewise, at a microscopic scale, short diffusion time may make water molecules to have less chance to experience their environment. In all cases, the recently reported findings [37,39] clearly show that there is a need to routinely provide diffusion time (and TE) when reporting and interpreting IVIM parameter values, both in preclinical and clinical settings. Our future work would then be to extend the present work to the case of multiple diffusion times and ultimately establish the relationship between the observed DW signals (as well as IVIM parameters) and the underlying organ (or tissue) microstructure inside the voxel.

## Conclusions

When using a sufficient number of low b-values, both BSP and LSQ generate close IVIM parameters (*D*, *F* and D*) to the ground-truth in the simulation case. Nevertheless, BSP generates better IVIM parameter maps than LSQ by generating fewer outliers, lower RE and higher SSIM. However, if the image is corrupted heavily by noise, the performance of BSP will more rapidly degrade and begin to rely on the number of low b-values. Nevertheless, even in this case, BSP is still more performant than LSQ. In contrast, LSQ is less sensitive to noise by generating rather stable IVIM parameter maps. Without using low b-values, neither LSQ nor BSP will be able to give a meaningful estimation of IVIM parameters.

## Supporting information

S1 Data In vivo and simulated data. (ZIP)
